# Histological and Histomorphometric Insights into Implant Bed Preparation: A Systematic Review

**DOI:** 10.3390/jcm14134538

**Published:** 2025-06-26

**Authors:** Piotr Kosior, Sylwia Kiryk, Agnieszka Kotela, Jan Kiryk, Julia Kensy, Marzena Laszczyńska, Mateusz Michalak, Jacek Matys, Maciej Dobrzyński

**Affiliations:** 1 Department of Conservative Dentistry with Endodontics, Wroclaw Medical University, Krakowska 26, 50-425 Wroclaw, Poland; 2 Department of Pediatric Dentistry and Preclinical Dentistry, Wroclaw Medical University, Krakowska 26, 50-425 Wroclaw, Poland; s.roguzinska@gmail.com (S.K.); maciej.dobrzynski@umw.edu.pl (M.D.); 3 Medical Center of Innovation, Wroclaw Medical University, Krakowska 26, 50-425 Wroclaw, Poland; kotela.agnieszka@gmail.com (A.K.); marzenalaszczynska@gmail.com (M.L.); mateusz.michalak92@gmail.com (M.M.); 4 Dental Surgery Department, Wroclaw Medical University, Krakowska 26, 50-425 Wroclaw, Poland; jan.kiryk@umw.edu.pl; 5 Faculty of Dentistry, Wroclaw Medical University, Krakowska 26, 50-425 Wroclaw, Poland; julia.kensy@student.umw.edu.pl

**Keywords:** implant bed preparation, implant site preparation, histological analysis, histomorphometric, histology

## Abstract

**Objective:** To assess the bone histological changes and histomorphometric parameters when using different implant site preparation methods. **Methods:** A systematic search was conducted in March 2025 across the PubMed, Scopus, and Web of Science (WoS) databases following the PRISMA guidelines. An initial search of the databases yielded 338 potentially relevant articles. Ultimately, a total of 29 articles were included in the qualitative synthesis in this review. The considerable heterogeneity among the included studies precluded a meta-analysis. **Results:** This systematic review showed that, among all the assessed implant site preparation methods, which were drilling, laser, piezoelectric surgery, osteotomy and osteodensification, the classical drilling method was more likely to cause adverse changes at the drill site, such as microcracks, uneven bone margins, osteocyte damage and thermal injury. In contrast, alternative methods resulted in fewer microcracks, minimal inflammation, a reduced risk of thermal tissue damage and denser, more regular bone formation. When using these methods, the %BIC parameter was higher than when using the drilling method. **Conclusions:** Using alternative techniques to prepare the implant bed creates favourable conditions for proper healing and osseointegration by eliminating defects resulting from the drilling method. However, it should be noted that satisfactory results can be achieved using the classical method if the correct parameters of the drill rotation, cooling and load are employed. Further studies based on a uniform methodology are necessary to determine the most efficient and safest parameters for each method.

## 1. Introduction

Modern dentistry offers various methods for replacing missing teeth, ranging from removable dentures to permanent solutions such as crowns and bridges, as well as prosthetic reconstructions supported by implants [1,2,3]. Replacing lost teeth is essential for restoring the proper function of the masticatory system, maintaining or improving the aesthetics of the smile and face, and preventing the negative consequences of tooth loss [4,5,6]. The most advanced and convenient method for replacing missing teeth is currently implant-supported reconstruction. This approach can be used to treat partial tooth loss, where implants support crowns or bridges, as well as in cases of edentulism, where they support complete dentures [7,8,9]. This form of treatment eliminates the discomfort associated with the instability of removable dentures and their negative impact on periodontal tissues during use. When creating a prosthetic bridge, implant-supported reconstruction also avoids the need to grind down healthy teeth to serve as abutments [10,11]. For a dental implant to function properly, it must undergo osseointegration, which is essential for achieving stability within the bone and ensuring long-term retention of the prosthesis. This process is influenced by numerous factors, including the implant’s design, the materials used, the biocompatibility, the placement technique, and the preparation of the implant site [12,13].

**Figure 1:**
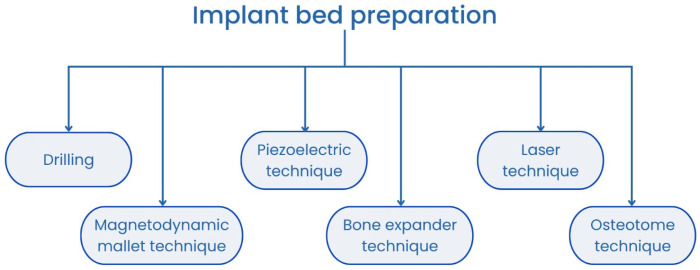
Methods for implant bed preparation.

The placement of a dental implant is preceded by the preparation of the implant site (bed) [14]. Before the implant can be inserted into its target location, the bone must be shaped and sized appropriately. Proper site preparation significantly increases the likelihood of successful osseointegration and long-term implant stability [15,16,17]. The most common methods for preparing the site for a dental implant include conventional drilling, piezoelectric techniques, laser, osteotome techniques, magneto-dynamic mallet techniques, and bone expander techniques (see Figure 1) [18,19,20]. Among these, drilling remains one of the most widely used techniques. With this method, the correct selection of the rotation speed and cooling parameters is critical for achieving the desired outcome [18,21,22,23]. An alternative method that yields results comparable to drilling is the use of a piezoelectric device. Studies have shown that this technique results in less postoperative pain and swelling, as well as improved implant stability [19]. Similar advantages have been observed with the use of the Er,Cr:YSGG laser. In addition to its antibacterial properties, this laser induces ablation during the procedure, producing less bone dust [18]. In cases involving low-density bone, the osteotome technique can enhance the bone quality and thereby improve the implant stability [24]. Another notable method is the use of a magneto-dynamic mallet. According to Baldi et al., this technique provides greater precision in terms of site preparation compared to conventional drilling and causes less trauma to the bone during the procedure [25].

The process of implant bed site preparation induces a range of histological changes in bone tissue. The alterations primarily result from mechanical and thermal trauma [21,26,27,28,29]. Control of the bone temperature during osteotomy is of paramount importance, as exposure to temperatures near 47 °C for a duration of one minute is widely acknowledged as the critical threshold leading to irreversible osteonecrosis [30,31,32]. Histological evaluations in human and animal models have demonstrated the presence of bone chips, microcracks, irregular bone surface, areas of osteonecrosis, bone remodeling, inflammatory cells and disruption of the trabecular architecture [33,34,35]. These changes are closely associated with the techniques for implant bed site preparation and the drilling parameters, including the rotational speed and irrigation efficiency [36,37]. Structural defects in the peri-implant region can interfere with the process of osseointegration. These disruptions can weaken the implant stability and, in more severe cases, lead to implant failure or rejection. Techniques that preserve the bone microstructure and cellular viability appear to promote more favorable healing environments, underscoring the importance of atraumatic surgical protocols in optimizing osseointegration.

The existing dental literature on implant site preparation is extensive; however, a comprehensive analysis of the histological outcomes following different techniques for implant bed site preparation is still missing. To date, no systematic review has carefully examined the microscopic bone response to various osteotomy methods. This topic is important because histological analysis provides information about bone vitality, healing processes, and the biological consequences of mechanical interventions during implantation. The primary aim of this review is to answer the following research question: Which implant site preparation techniques lead to the least histological changes and trauma to bone tissue? By evaluating the histological parameters, this study aims to clarify the biological impact of surgical choices. These findings could help develop better clinical guidelines and support the use of less invasive and biologically respectful methods in dental implantology.

## 2. Materials and Methods

### 2.1. Focused Question

This systematic review followed the PICO framework as follows: In patients receiving dental implants (population), do alternative implant bed preparation techniques (investigated condition) result in improved histological and histomorphometric outcomes (outcome) compared to conventional drilling techniques (comparison condition)?

### 2.2. Protocol

**Figure 2:**
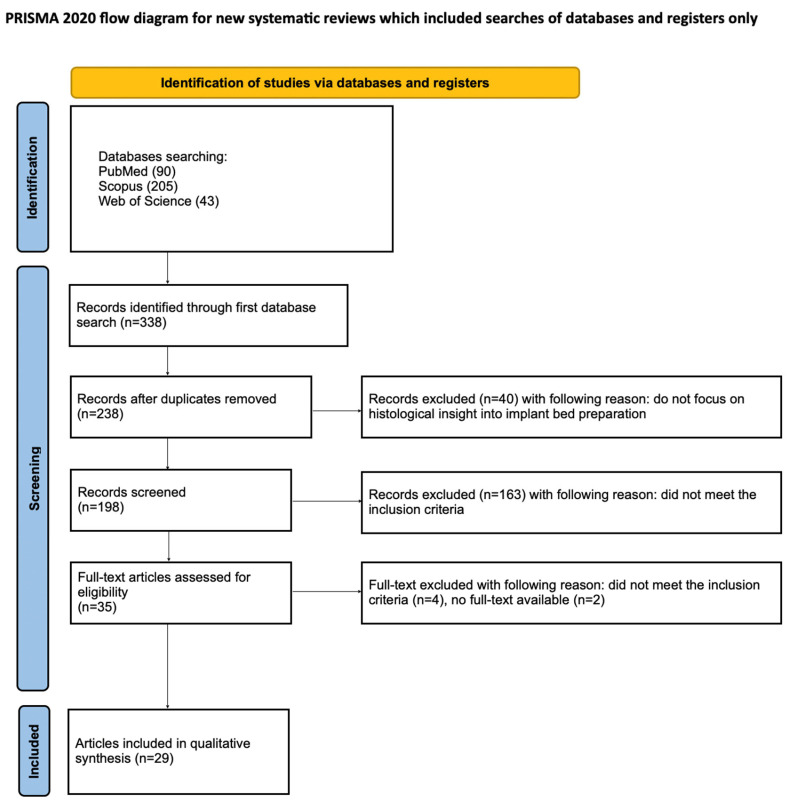
PRISMA flow diagram.

The article selection process for the systematic review was systematically detailed using the PRISMA flow diagram (Figure 2). The systematic review was registered with the Open Science Framework under the following link: https://osf.io/eytxu (accessed on 20 May 2025).

### 2.3. Eligibility Criteria

The researchers decided to only include articles that fulfilled the following criteria:

- Studies focusing on histological analysis or histomorphometric analysis of an implant bed;
- In vitro studies;
- In vivo;
- Studies in English;
- Non-randomized controlled clinical trials (NRSs);
- Randomized controlled clinical trials (RCTs).

The reviewers established the following exclusion criteria:

- Studies not focusing on histological or histomorphometric insight into implant bed preparation;
- Non-English papers;
- Clinical reports;
- Editorial papers;
- Review articles;
- Systematic review articles;
- No full-text accessible;
- Duplicated publications.

No restrictions were applied with regard to the year of publication.

### 2.4. Information Sources, Search Strategy, and Study Selection

A comprehensive literature search was carried out in March 2025 across the PubMed, Scopus, and Web of Science (WoS) databases to find studies that met the established inclusion criteria. To narrow the focus to the histological and histomorphometric aspects of implant site preparation, the search strategy was confined to titles and abstracts containing the following keywords: “implant bed preparation” OR “implant site preparation” AND “histologic” OR “histomorphometric” OR “histology.” The selection process adhered strictly to the eligibility guidelines, and only studies with accessible full-text versions were considered for inclusion.

### 2.5. Data Collection Process and Data Items

Six independent reviewers (M.L., J.K., A.K., S.K., J.K., and M.M.) independently screened and selected studies that fulfilled the inclusion criteria. The data extracted from each article included the first author’s name, year of publication, study type, article title, details of the implant site preparation technique, and associated histological and histomorphometric findings. All the collected information was systematically documented in a standardized Excel spreadsheet.

### 2.6. Assessing Risk of Bias in Individual Studies

In the initial stage of study selection, all the reviewers independently evaluated the titles and abstracts to minimize the risk of bias. The inter-reviewer consistency was measured using Cohen’s kappa statistic. Any conflicts concerning the inclusion or exclusion of specific studies were addressed and resolved through group discussion among the authors.

### 2.7. Quality Assessment

Two blinded reviewers (J.M. and M.D.) independently evaluated the methodological quality of each included study using the Joanna Briggs Institute (JBI) checklist for quasi-experimental designs (non-randomized studies). This assessment tool comprises nine targeted questions designed to appraise the procedural rigor of such studies.

- Is it clear in the study what is the “cause” and what is the “effect”?
- Were the participants included in any similar comparisons?
- Were the participants included in any comparisons receiving similar treatment/care, other than the exposure or intervention of interest?
- Was there a control group?
- Were there multiple measurements of the outcome both before and after the intervention/exposure?
- Was a follow up completed, and if not, were the differences between groups in terms of their follow up adequately described and analyzed?
- Were the outcomes of the participants included in any comparisons measured in the same way?
- Were the outcomes measured in a reliable way?
- Was an appropriate statistical analysis used?

Each item on the checklist could be marked as “yes,” “no,” “unclear,” or “not applicable.” In cases where the reviewers provided differing answers, discrepancies were resolved through discussion to reach a consensus. The inter-rater reliability was evaluated using Cohen’s kappa statistic, calculated with MedCalc software (version 23.1.7; MedCalc Software Ltd., Brussel, Belgium). The analysis yielded a kappa coefficient of 0.85 (*p* < 0.001), reflecting a high level of agreement and indicating near-perfect consistency between reviewers.

## 3. Results

### 3.1. Study Selection

The initial search of the PubMed, Scopus, and WoS databases yielded 338 potentially relevant articles. After removing duplicates, 238 articles remained for screening. After an initial search of the titles and abstracts, 203 articles that did not meet the inclusion criteria were excluded. Of the remaining 35 studies, the full text could not be accessed for two studies and four were excluded because no histological or histomorphological analysis was performed in them. Ultimately, a total of 29 articles were included in the qualitative synthesis in this review. The considerable heterogeneity of the included studies prevented the conducting of a meta-analysis.

### 3.2. General Characteristics of the Studies Included in the Review

Among the included papers, only Tabrizi et al. [38] conducted studies on human subjects. The remaining studies were performed using animal models. Eight studies were conducted on pigs [39,40,41,42,43,44,45,46], utilizing various anatomical sites, such as the tibia [42,43,46], rib [44], mandible [41,45], maxilla [39], and calvaria [40]. Seven studies involved the mandibles of dogs [47,48,49,50,51,52,53]. Another seven papers used sheep models [54,55,56,57,58,59,60], including four that targeted the iliac crest [55,56,57,59], two that focused on the mandible [54,58], and one that used the femoral head [60]. Two studies placed implants in bovine ribs [61,62], while one study used rabbit tibias [63]. Sirolli et al. examined rat tibias [64], and Chen et al. used the maxillae of rats [65]. Among the various implant site preparation techniques analyzed, the most frequently used method was drilling [38,39,40,41,43,44,45,46,47,48,49,50,51,52,53,54,55,56,57,58,59,60,61,62,63,64,65,66]. However, several authors also employed piezoelectric devices [40,42,46,48,51,57,59,61,64] or the Er:YAG laser [41,50,57,60,62,66]. Notably, Schierano et al. used a magneto-dynamic mallet [43], distinguishing their approach from other studies. There was considerable variability in the healing times across studies. Some researchers evaluated the bone response immediately after implantation [39,43,44,45,60,63,65]; meanwhile, in another case, the healing period was evaluated up to four months [52] (see Table 1).

### 3.3. Main Study Outcomes

The objective of this review was to assess the histological and histomorphometric outcomes following the use of different implant bed preparation techniques. The main finding is that alternative methods, including piezosurgery, osseodensification, and laser-assisted techniques, achieve comparable or even superior bone healing and osseointegration compared to conventional drilling. Although various preparation methods were employed across the included studies, the healing results and measured histological parameters varied depending on the technique and experimental conditions. A detailed summary of the characteristics of the included studies is presented in Table 2.

#### 3.3.1. Conventional Burs

Conventional rotary drills have long been the most commonly used instruments for implant bed preparation. However, numerous studies have shown that, although effective, this technique can cause both mechanical and thermal trauma to the bone [38,44,55,61]. Histological evaluations have revealed microcracks, irregular bone surfaces, and the accumulation of debris and blood cells at drill-prepared sites, all of which may delay or impair the healing process [48,61]. Furthermore, high-speed drilling without adequate irrigation significantly increases the thermal damage, resulting in reduced bone viability [38,44]. Studies have also indicated that combining high-speed drilling with greater drilling depth worsens the loss of viable bone cells [38].

Nonetheless, bone viability can be preserved—and comparable healing outcomes achieved—by optimizing the drilling parameters, such as using low-speed drilling with sufficient irrigation [47,63,65]. Modifications to the drilling protocols, including the use of undersized osteotomies [53] and minimizing the lateral pressure [52], have been investigated to enhance the primary stability and improve the implant integration. However, delayed placement protocols and variations in the drill diameter produced inconsistent effects on bone remodeling. Some studies suggest that excessive compression or overheating during drilling may lead to microcracks and subsequent bone resorption [40,45,61].

#### 3.3.2. Piezosurgery

Piezosurgery has emerged as a technique that offers enhanced biological outcomes compared to conventional rotary instruments. Multiple studies have consistently shown that piezoelectric devices produce smoother and more uniform osteotomy sites, preserve osteocyte viability, and elicit reduced inflammatory responses [42,48,59,61]. Histological evaluations revealed that bone surfaces treated with piezosurgery exhibited fewer microcracks and better structural preservation, facilitating faster and more organized new bone formation, particularly in cancellous bone regions [64].

However, thermal damage to tissues has been reported when excessive force is applied during piezosurgery, emphasizing the need for precise surgical technique and careful load control [40]. The healing dynamics also vary between piezosurgery and traditional drilling. While rotary instruments tend to promote earlier, more organized osteogenesis, piezosurgery has been associated with more diffuse and widespread bone regeneration during later healing stages [46].

Overall, piezoelectric surgery demonstrates favorable biological effects and may be especially beneficial in anatomically delicate or low-density bone areas.

#### 3.3.3. Osseodensification

Osseodensification, a technique that compacts rather than removes bone tissue during osteotomy, has demonstrated promising histomorphometric outcomes. Studies have shown that this approach increases the bone density, particularly in the coronal region of the implant site, and supports the formation of new bone with a distinct granular microstructure. Although the differences in the bone-to-implant contact percentages compared to conventional drilling were not statistically significant, the enhanced peri-implant bone quality and improved mechanical stability suggest that osseodensification may be particularly beneficial in clinical cases requiring high primary stability, such as in low-density bone. Additionally, this method appears in one study to reduce micromovements and the risk of early implant failure [56].

#### 3.3.4. Laser

Laser-assisted osteotomy, particularly using Er:YAG lasers, presented significant advantages in minimizing the thermal and mechanical trauma to bone during implant site preparation [50,60,62,66]. Histological evaluations revealed that laser-prepared sites exhibited cleaner osteotomy walls, absence of trabecular collapse, and better preservation of bone microarchitecture compared to conventional drilling [60,62,66]. Early bone healing was faster in laser-prepared sites, with significantly higher bone-to-implant contact percentages observed at both the 3-week and 3-month intervals [66]. However, the early healing around laser-prepared implants sometimes showed wider peri-implant gaps, although these differences resolved with maturation, leading to comparable or superior osseointegration at later stages [50,57]. Notably, the choice of laser settings significantly influenced the outcomes: using the QSP mode minimized the thermal side effects and promoted better bone preservation compared to the MAX mode [41]. Although laser osteotomy requires a longer surgical time compared to conventional drilling, the biological benefits suggest it could be a valuable alternative, particularly in cases where preservation of the bone structure is critical [41,60,62].

#### 3.3.5. Osteotomy with Bone Condensation

Osteotomy techniques that rely on bone condensation rather than removal—such as the use of osteotomes and magneto-dynamic mallets—have shown positive effects on the peri-implant environment. Nkenke et al. [39] reported that osteotome techniques resulted in higher early bone-to-implant contact percentages compared to conventional drilling, particularly within the first three months of healing. Similarly, site preparation using a magneto-dynamic mallet led to increased bone formation, elevated osteoblast activity, enhanced peri-implant bone density, and improved primary implant stability [42].

These bone-condensing methods appear especially beneficial in areas of low bone quality, as they contribute to stronger mechanical anchorage and reduce the likelihood of peri-implant fibrous tissue formation. However, their effectiveness in dense bone remains less consistent, and the technique’s sensitivity necessitates careful execution to avoid unintended microdamage.

#### 3.3.6. Bone Healing and BIC Outcomes in Histological and Histomorphometric Evaluations

The histological assessment of the healing time varied significantly depending on the implant bed preparation technique used.

Conventional rotary drilling, the most frequently used technique, was associated with moderate healing dynamics. The histological sections often revealed irregular bone surfaces, microcracks, and damaged osteocytes, especially when higher drilling speeds and inadequate cooling were applied [38,55,61,63]. Bone-to-implant contact (BIC) and new bone formation were generally evident after 3 to 8 weeks [45,47,48], but complete organization of the newly formed bone often required 2–3 months or more [41,52,65].

In contrast, piezoelectric surgery showed more favorable biological outcomes. Several studies reported smoother osteotomy walls, better osteocyte viability, and early signs of organized bone formation, particularly in cancellous bone regions [42,46,48,61,64]. While osteogenesis following piezosurgery tended to be more diffuse in the early stages, the quality of the newly formed bone improved over time, becoming comparable or superior to conventional drilling by 30–60 days [57,59].

Er:YAG laser osteotomy produced variable results. When used with the proper settings, it preserved the bone microarchitecture and caused minimal thermal damage [50,60,62,66]. Some studies showed delayed but continuous bone formation, with mature lamellar bone surrounding the implant by 12 weeks [50]. However, improper load application during laser use could lead to thermal effects and alterations in the cortical bone [40,41].

Osteotome techniques, which rely on bone condensation rather than removal, demonstrated early signs of bone integration. The BIC values were notably higher in the first 1–3 months compared to drilling, especially in low-density bone [39]. Similarly, the magneto-dynamic mallet technique achieved a significantly larger area of newly formed bone, an increased osteoblast count, and lower amounts of fibrous tissue as early as 14 days post-implantation [42].

Osseodensification, although not significantly different in terms of the BIC values compared to drilling, resulted in increased bone density and enhanced coronal bone formation with a distinct granular structure. These findings suggest improved mechanical stability and better quality of peri-implant bone at the early healing stages (up to 2 months) [56].

Taken together, these findings indicate that preparation techniques involving bone preservation or condensation (such as piezosurgery, osteotomes, or magneto-dynamic mallets) tend to support faster and more favorable biological healing responses compared to conventional drilling, particularly in cancellous or low-density bone regions (see Table 3).

### 3.4. Quality Assessment of Included Studies

For all of the nine questions, 19 papers received a positive answer to nine of them [43,45,46,47,48,49,50,51,52,53,55,56,57,58,59,60,64,65,66] and 10 papers received a positive answer to eight of them [38,39,40,41,42,44,54,61,62,63] (see Table 4).

## 4. Discussion

The aim of this systematic review was to evaluate the histological and histomorphometric outcomes of various implant bed preparation techniques. The studies included in the review utilized several preparation methods, including conventional rotary drilling [38,39,40,43,44,45,46,47,48,49,50,51,52,53,54,56,57,58,59,60,61,62,63,64,65,66], piezoelectric instrumentation [40,42,46,48,51,57,59,61,64], Er:YAG laser osteotomy [41,50,57,60,62,66], osteotome-based bone condensation [39,43], and the osseodensification technique [56]. The experimental models were primarily based on animal studies, involving pigs [39,40,41,42,43,44,45,46], dogs [47,48,49,50,51,52,53], sheep [54,55,56,57,58,59,60], cattle [61,62], and rabbits [63]. Only one study was conducted in human subjects [38]. The results suggest that conventional drilling is associated with a higher incidence of adverse effects, including microcracks in the bone tissue [48,61,62], irregular osteotomy surfaces [60,61], thermal damage [38,44], and reduced bone-to-implant contact (%BIC) values [39,41,48,56,66]. In contrast, alternative methods such as piezoelectric surgery, laser-assisted osteotomy, osseodensification, and osteotome-based techniques demonstrated comparable or improved %BIC values [39,48,57,66]. These techniques were also associated with favorable bone compaction [42], a reduced incidence of microcracks [60,61,62], lower thermal injury to the bone structures [40,50,60,62], a diminished inflammatory response [42,59], and better structural organization and quality of the newly formed bone tissue [41,43,56,59,61,62,64,66]. Overall, this review indicates that implant bed preparation methods other than conventional drilling may provide more biologically favorable conditions for healing and support more effective osseointegration of dental implants.

Conventional drilling remains one of the most commonly used techniques for preparing bone tissue prior to implant placement. Among the 29 studies included in this review, 28 evaluated the histological and histomorphometric outcomes following the use of rotary drills [38,39,40,41,43,44,45,46,47,48,49,50,51,52,53,54,55,56,57,58,59,60,61,62,63,64,65,66]. Only one study [42] did not utilize this method. The findings of most studies consistently indicate that, compared to alternative techniques, conventional drilling is associated with the highest risk of disrupting bone healing and impairing osseointegration. A key factor contributing to this is thermal trauma to the bone. As shown by Tabrizi et al. [38] and Kosior et al. [44], drilling at excessive rotational speeds and with inadequate irrigation significantly reduces the bone vitality, especially when combined with an increased drilling depth [38]. To minimize this risk, the use of low-speed drilling is recommended. According to Kosior et al. [44], speeds below 800 rpm yielded favorable histological outcomes across different drilling systems and irrigation conditions. Moreover, studies have demonstrated that implant bed preparation can be successfully performed at very low rotational speeds even in the absence of irrigation [47,63,65]. The irrigation method itself was another critical variable affecting the bone response. Trisi et al. [54] demonstrated that internal or combined (internal and external) irrigation methods are most effective in reducing the thermal changes. Additionally, Stelzle et al. [40] observed that increasing the axial load during drilling led to greater thermal alterations in the bone, even at forces lower than those applied during piezoelectric osteotomy. Mechanical trauma was another common complication associated with rotary drilling. Histological findings revealed the presence of microcracks, damaged osteocytes, irregular osteotomy surfaces, and bone debris, all of which were observed more frequently with drills than with alternative methods such as piezoelectric devices, osseodensification, or laser osteotomy [48,61,62]. Furthermore, the newly formed bone in drill-prepared sites tended to be less dense, more irregular, and less mineralized, and it exhibited a higher incidence of necrotic areas and reduced bone volume [41,43,56,57,61,66]. These histological and histomorphometric alterations may negatively affect both the speed and the quality of bone healing, ultimately compromising the success and long-term stability of dental implants.

Nevertheless, it is important to note that improved healing of the cortical bone was observed with the conventional drilling method compared to the piezoelectric technique [64]. This finding highlights the need to consider the local bone characteristics when selecting the implant bed preparation method. The use of piezoelectric devices was evaluated in 9 out of the 29 studies included in this review [40,42,46,48,51,57,59,61,64], while 20 studies did not incorporate this technique [38,39,41,43,44,45,46,47,49,50,52,54,55,56,58,60,62,63,65,66]. Piezosurgery has been consistently shown to cause less mechanical and thermal damage to the bone compared to conventional drilling. Osteotomy walls prepared using piezoelectric instruments tend to be smoother, exhibit fewer microcracks, and show more regular and undistorted histological architecture [61,64]. Additionally, this technique has been associated with a reduced inflammatory response, an absence of osteoclasts, and a high density of osteoblasts in the healing area [42,59]. Residual bone debris is also absorbed more rapidly in piezosurgery-treated sites compared to those prepared using rotary drills [48]. Collectively, these findings suggest that piezoelectric preparation is less invasive and creates a biologically favorable environment for implant healing, offering high potential for successful osseointegration. Notably, better healing outcomes were observed in cancellous bone when using piezoelectric instruments, in contrast to conventional drilling methods [64]. This makes the technique particularly advantageous in cases involving fragile or low-density bone structures. Furthermore, piezoelectric tools significantly reduce the risk of thermal injury to the bone. However, heat-related damage may still occur if excessive force is applied during the procedure [40]. Sakita et al. [46] reported that, although drilling resulted in a greater initial quantity of new bone, mature bone tissue ultimately developed after using both preparation methods over time.

The Er:YAG laser method prepares bone without direct contact, which reduced the thermal damage and preserved the trabecular structure at the margins of the osteotomy sites [50,60,62]. The bone-to-implant contact (BIC) values in laser-prepared sites reached up to 74.51% [57], comparable to the values achieved with piezoelectric techniques. In addition, laser preparation resulted in cleaner cavities and better preservation of the bone structure, which promoted faster healing [60,62,66]. The Er:YAG laser enhanced the bone healing and implant integration by supporting the formation of organized new bone, reducing the inflammatory response, and maintaining the bone’s natural architecture [57]. Likewise, histological analysis of implant sites prepared using the osteotome technique showed a favorable peri-implant bone response. Higher BIC values were observed on the palatal side of implants loaded immediately or after 1 to 3 months of healing, with percentages reaching up to 87% [39]. This outcome was likely related to the lateral condensation of trabecular bone achieved during osteotome use, which improved the primary stability and stimulated early bone apposition. These findings indicated that controlled bone compaction facilitated early osseointegration, particularly in the maxillary bone, which typically exhibited lower bone density [39].

Osseodensification (OD) is a technique for implant site preparation that has demonstrated promising outcomes in promoting bone healing and enhancing implant integration. Histological analyses of implant sites prepared using OD burs have shown a significantly greater bone volume (%BV) around the implants—approximately 30% higher—particularly in regions characterized by low bone density [56]. OD instruments are engineered to compact rather than remove bone, thereby preserving the trabecular architecture and increasing the number of nucleation sites for mineral deposition. These features indicate active bone formation and suggest potential for long-term densification of peri-implant bone tissue. Furthermore, studies have reported an improvement in the bone-to-implant contact (BIC) ratio with osseodensification (49.58%) compared to conventional drilling (46.19%) [56], indicating enhanced mechanical interlocking and stability. These findings support both the primary and secondary stability of the implants, which are critical for predictable healing and long-term clinical success.

It is worth noting that despite the continuous expansion of the possibilities of using lasers among the qualified works, the only laser used was the Er:YAG laser. There is room here for research using other lasers and performing histological and histomorphological studies. Perhaps this will increase the number of available techniques for preparing implant beds. Despite the significant advancements in implant bed preparation techniques, each method presents specific limitations that warrant further investigation. The majority of the current evidence is derived from in vitro experiments or animal studies, which limits the direct applicability of these findings to clinical practice. Consequently, there is a clear need for well-designed, prospective clinical trials involving human subjects to validate and expand upon these results. It should also be noted that besides the preparation of the bone, the implant surface and geometry also play an important role in osseointegration and influence the BIC [67,68]. Researchers also noted that the wear of drills used to prepare the implant site may affect the quality of the prepared beds and thus the osteointegration of the implants [69]. However, there are no histological or histomorphological studies assessing this effect. Therefore, further studies should take into account the cumulative effect of all these factors. Moreover, considerable variability exists among the available studies in terms of the study design, sample size, follow-up duration, and evaluation methodologies. This heterogeneity poses challenges in comparing the outcomes across studies and hampers the development of standardized protocols that could be reliably implemented in clinical settings. Future research should prioritize long-term clinical outcomes, employ standardized assessment criteria, and incorporate advanced imaging technologies to gain deeper insight into the biological responses elicited by each preparation method. Such efforts are essential not only to improve the implant success rates but also to enable the customization of surgical approaches tailored to individual patient anatomy and bone quality.

## 5. Conclusions

This systematic review of twenty-nine studies provides valuable insights into the histological and histomorphometric outcomes of various implant bed preparation techniques. Alternative methods—such as piezosurgery, laser-assisted techniques, osteotomes, and osseodensification—generally offer more favorable biological conditions for healing and implant integration compared to conventional drilling. They are associated with fewer microcracks, smoother osteotomy walls, reduced thermal and inflammatory responses, and better bone organization and density. However, these techniques are not universally superior. Conventional drilling has shown better results in cortical bone, while piezosurgery and condensation-based methods may be more suitable for low-density bone. The clinical success of any technique depends heavily on the use of appropriate parameter settings and the bone quality. Therefore, future research should focus on clinical validation, long-term outcomes, and methodological standardization to support evidence-based selection of implant site preparation methods.

## Figures and Tables

**Table 1:** General characteristic of the studies.

Study	Aim of the Study	Material and Methods	Results	Conclusions
Nkenke [39]	Analysis of histomorphometric parameters of implants implanted in the maxilla of minipigs.	In 9 mini-pigs, 4 teeth were extracted and 6 implants were placed in the maxilla, which were immediately or after 1, 2, 3, 4 and 5 months loaded with dentures. The implant beds were prepared using osteotomies or spiral drills. After 6 months of loading, the implants were removed, sectioned and the image digitally analyzed.	The palatal BIC was higher for the osteotome technique with healing up to 3 months, while it was higher for the drill with healing up to 4–5 months. The longer the healing time, the greater the resorption, regardless of the preparation technique used.	Healing time and implant site preparation technique influence histomorphometric parameters such as BIC and bone resorption.
Stubinger [57]	To assess how different implant site preparation techniques affect the osseointegration of titanium dental implants.	108 titanium implants were placed in the pelvis of 6 sheep. Implant sites were prepared using a Er:YAG laser, piezoelectric device or conventional drills. After 4, 6 or 8 weeks, the BIC was assessed histologically, and implant stability was evaluated. Fluorochrome labeling with calcein green and xylenol orange was used to visualize bone remodeling.	Initial BIC values (4 weeks) were slightly higher in laser and piezoelectric groups versus drill osteotomy. At 6 weeks, the BIC decreased in the laser and piezoelectric groups but increased in the drill group. By 8 weeks, BIC improved in laser and piezoelectric groups. Removal torque testing showed the laser group had the highest values at 8 weeks. Fluorescence labeling demonstrated active bone remodeling in all groups.	Laser and piezoelectric osteotomy techniques showed comparable or superior results to conventional drilling for dental implant placement. Both alternative methods demonstrated favorable osseointegration by 8 weeks, with laser osteotomy achieving significantly higher mechanical stability.
Gaspar [63]	Comparison of histological changes after implant osteotomy preparation using low -speed drilling without irrigation and high-speed drilling with irrigation.	36 implants were inserted into the tibias of the rabbits using 2 types of preparation speeds (low without irrigation and high with irrigation) using drills of increasing diameter. Samples were taken for histological analysis.	Histological analysis revealed no differences between the two surgical drilling techniques.	Both types of drillings similarly preserve bone cell viability, allowing the surgeon to choose the method based on other factors.
Scarano [61]	Comparison of two methods of bone preparation for implantation: piezoelectric and using a drill	In 10 beef ribs, 50 bone perforations were made using a drill and 50 using a piezoelectric device. The samples were sectioned and evaluated using a light microscope.	Bone preparation with a piezoelectric device resulted in a bone structure with fewer microcracks, a more regular structure of the bone edges and a histological image of the bone structure that was less disturbed compared to the method using a drill.	Bone prepared for implantation using a piezoelectric device has a more desirable structure than when using a drill.
Sakita [46]	Comparison of histomorphological aspects of bone tissue after osteotomy of porcine tibias using a piezoelectric system and rotary instruments, taking into account different postoperative periods.	After exposing the tibia in pigs, 4 preparations for dental implants were performed: 2 using classic drills, where the widest one had a diameter of 3 mm, and the others using a piezoelectric system. Then, the wounds were sutured. The animals were killed after 2, 7, 14 and 28 days.	After 2 days, the tissue edges were jagged after piezosurgery and smooth after using rotary instruments. After 7 days, greater osteogenesis was visible after using classical drills. After 14 days, the piezo group showed diffuse bone formation throughout the surgical site, with a vascular stroma composed of adipose tissue, the presence of bone trabeculae with osteocytes, and a peripheral line. In the rotational group, there was intensive neoformation of the 1/3 of the cortical and medullary bone starting with thicker trabecular bone in greater quantity and better organization.	Bone neoformation was more evident when the implant site was prepared using conventional rotary instruments.
Kesler [66]	To evaluate whether implant site preparation with an Er:YAG laser improves implant osseointegration.	The implantation site was prepared using an Er:YAG laser (test group) in 18 rats and a drill (control group) in 18 rats. Implant samples with surrounding bone were examined after 3 weeks or 3 months. %BIC was assessed.	%BIC after 3 weeks and 3 months was higher for the test group (Er:YAG laser).	Preparation of the implant site using an Er:YAG laser increases the successful implant osseointegration.
Semez [62]	To compare the histological effects of the Er:YAG laser and conventional tools on tissues during implant bed preparation.	The bovine tongue and rib were used for the study. A 5 mm Er:YAG laser scalpel and a conventional bur were used to prepare the holes. Each group consisted of six laser-prepared samples and one (soft tissue) or six (hard tissue) control samples. The tissues were fixed in 10% formalin, processed in ethanol, xylene, and paraffin, and analyzed microscopically after staining.	Implant bed prepared using conventional burs contains more debris and blood cells than laser treated samples. Thermal damage to soft tissue was observed with the laser’s MAX mode but not with the QSP mode.	Laser use for implant bone preparation is more promising in terms of successful osseointegration as it does not form debris in the implant bed.
Semez [41]	To evaluate the use of an Er:YAG laser for implant site preparation and reduce the time between primary and secondary stability, focusing on immediate occlusal loading in single implants.	The study included 6 minipigs that underwent implant site preparation using either a conventional bur kit or an Er:YAG laser. Four implants- positions 34, 37 (traditional burs) and 44, 47 (laser preparation) were placed in each pig. Bone samples were collected at 45, 60, 90 and 120 days post-surgery and subjected to the histological analysis.	Implants placed with the Er:YAG laser showed better bone healing and remodeling, especially in deeper areas, but with limited coronal bone-to-implant contact. In contrast, the bur-prepared sites had some implants fail due to necrotic or immature bone and weak coronal BIC. The laser promoted more consistent and less traumatic healing compared to the conventional bur method.	The use of the Er:YAG laser for implant site preparation results in higher-quality bone healing, with more lamellar bone and less necrosis compared to conventional drilling.
Schwarz [50]	To assess how using an Er:YAG laser for implant bed preparation affects the osseointegration of titanium dental implants compared to conventional drilling methods.	Implant sites in the lower jaw of 4 beagle dogs were prepared using either an Er:YAG laser or conventional drills. 24 implant channels were created, with 3 different commercial titanium implants randomly inserted into both sites. After healing periods of 2 and 12 weeks, the histological assessment was performed.	The Er:YAG laser preparation showed no thermal damage but created significantly wider peri-implant gaps and lower initial bone-to-implant contact at 2 weeks compared to conventional drilling. By 12 weeks, both methods achieved comparable osseointegration with mature lamellar bone formation, despite the laser method requiring significantly more preparation time.	Er:YAG laser could be a promising instrument for implant bed preparation.
Zeitouni [60]	Comparison of implant bed preparation: Er:Yag laser vs. conventional drilling technique.	Micro-computed tomographic histomorphometric analysis and histological examination were performed after bed preparation using different laser parameters and the conventional dental drill technique in the ovine femoral heads.	There was absence of trabecular collapse and reduced thermal and mechanical damage with Er:Yag laser preparation.	Laser cutting promotes tissue healing, unlike conventional drilling, which may delay healing through trabecular collapse and thermal damage.
Fujiwara [48]	To evaluate the effect of two methods of implant bed preparation: using a drill or piezoelectric device on osseointegration and bone level.	In 12 dogs, implantation beds were prepared using a piezoelectric device (test group) and drills (control group). Samples were examined after 4 and 8 weeks using an Eclipse Ci microscope. The following were assessed: IS-B and M-B distance, BIC, pre-existing bone and soft tissue.	The amount of bone formation was greater at 8 weeks than at 4 weeks regardless of the used method. Mean distance between B and M after 4 and 8 weeks was smaller in the piezoelectric group.	Both tested methods provide similar and satisfactory histomorphological parameters, which allows for proper osseointegration.
Stelzle [40]	To compare piezosurgery and conventional drilling for implant site preparation.	360 implant beds were prepared on the porcine skull using a drill, trephine drill or piezoelectric device. The drills used external cooling, and the piezoelectric device used internal cooling. The samples were subjected to histomorphometric analysis. The following were examined: both sides of the cortical bone, the lateral walls of the prepared implant site on both sides and its bottom.	Thermal tissue changes increased with higher loads for both piezosurgery and trephine bur, with maximum effects at 900–1000 g for piezosurgery and 700–800 g for the trephine bur. The spiral bur showed the highest thermal effects at 400–500 g. The thermal changes were greatest in the cortical bone and a positive correlation was observed between load and thermal alterations for both techniques.	Piezosurgery causes significantly higher temperatures and more thermal tissue changes when the load exceeds 500 g and is slower compared to spiral bur and trephine bur for implant site preparation.
Schierano [42]	To investigate the early cellular and molecular processes involved in dental implant osseointegration when using piezoelectric surgery.	Two implant sites were created in the tibiae of four minipigs. After implantation cortical bone thickness and Implant Stability Quotient (ISQ) were measured. After 14 days CT scans and ISQ measurements were repeated. Bone sections were then processed for histological and biomolecular analyses.	48% of the tissue around the implants was newly formed bone, with a high presence of osteoblasts. The thickness of this new bone ranged from 300 to 1600 μm. No osteoclasts were detected, and inflammatory cell presence was minimal.	Piezoelectric surgery promotes a favorable biological response at implant sites, marked by increased osteogenic factors and new bone formation. These changes are associated with improved implant stability, as indicated by higher ISQ values.
Sirolli [64]	To investigate the influence of conventional drilling and piezosurgery on bone healing around implants.	15 male rats received titanium implants in their tibias, prepared with conventional drilling or piezosurgery. After 30 days, the histomorphometric evaluation was performed, comparing the BIC, bone area and mineralized tissue in cortical and cancellous regions.	Piezosurgery showed better bone healing in cancellous bone, with higher bone area and mineralized tissue percentage. Conventional drilling performed better in cortical bone, particularly for bone-to-implant contact.	The surgical preparation technique’s effectiveness varies depending on the bone region.
Zizzari [59]	Assessment of differences in bone tissue around dental implants after piezoelectric technique vs. conventional drilling technique preparation.	24 dental implants were implanted using 2 different preparation techniques into the iliac crest of sheeps. Samples were collected for analysis after 15 and 30 days.	No statistically significant higher expression of iNos and Bax when using the conventional drill technique	Piezoelectric bed preparation technique may reduce peri-implant inflammation and improve bone tissue healing
Trisi [56]	To assess a new method for preparing implant sites that could improve the bone density, ridge width, and the stability of implants after placement.	Implants were inserted into the iliac bones of two sheep, with one side prepared with standard drilling and the other using the osseodensification method. After a healing period of two months, the animals were euthanized and biomechanical and histological evaluation was performed.	The drilling group showed a thin layer of new bone with some broken trabeculae and fragments. The bone densification group showed thicker bone at the implant site, especially at the top, with more active bone formation and higher bone density. The drilling group had approximately 30% greater bone volume (%BV), while there was no significant difference in BIC.	The osseodensification technique increased bone volume around implants in low-density bone compared to traditional drilling, which may help improve implant stability and reduce micromovement.
Schierano [43]	To evaluate whether the magneto-dynamic mallet technique is more effective than conventional drilling.	12 titanium implants were placed in 3 male mini pigs tibias with half using each technique. Implant stability was measured immediately and after 14 days. Samples underwent histological and biomolecular assessment.	Mallet-prepared sites showed significantly better bone formation than drill sites, with higher bone percentage and osteoblast counts. The key findings include elevated BMP-4 expression, distinctive trabecular bone densification visible on CT around implants and elevated inflammatory markers, but balanced increased anti-inflammatory IL-10. Both techniques showed increased implant stability after 14 days.	The magneto-dynamic mallet technique effectively prepares sites for dental implants, creates more bone and osteoblasts than conventional drilling, which increases primary stability and promotes osteogenesis. This technique is less effective for dense bone but valuable for sites with thin cortex and poor bone quality.
Cesaretti [51]	Evaluation of soft and hard tissue integration around the implant using juxta or sub-crestally implantation. Comparison of site preparation using drills or sonic devices.	Implantation of two implants was performed at different bone depths in each of the six dogs. The test site was prepared 1.3 mm deeper than the control site. After 8 weeks, samples were taken for histological analysis.	The test sites showed greater bone loss and a more apical placement of the peri-implant mucosa.	The sub-crestal implant showed the greatest horizontal crestal bone loss. No significant differences between the bed preparation methods.
Trisi [54]	Evaluation of the effect of irrigation on implant osseointegration.	A total of 20 implants were implanted in the mandibles of 5 sheep. Depending on the cooling used during the procedure, they were divided into groups: A—no irrigation; B—internal irrigation; C—external irrigation; D—external and internal irrigation. After 2 months, histomorphometric and histological analysis was performed.	The studied parameters were more favorable in the groups in which irrigation was used, especially when internal or combined irrigation (external + internal) was used.	The presence of irrigation and its type have a significant impact on implant osseointegration.
Trisi [55]	Evaluation and comparison of two implant bed preparation protocols: single drill or drill sequence.	A total of 20 implants were implanted in 2 sheep. Half using 1 pilot drill and 10 implants using a drill sequence. After 2 months, the following parameters were assessed: %BIC, %BV, VAM, RT and ISQ.	In the test group, the studied histomorphometrical parameters were significantly higher. Osteocorticization and VAM was greater in the test group.	Implant placement using a drill sequence allows for better histomorphometric parameters and, as a result, better osteointegration of the implants.
Fujiwara [47]	Comparison of the effect on implant osseointegration of bed preparation with irrigation and high speed or without irrigation and low speed.	In 12 dogs, 3 months after tooth extraction, implantation sites were prepared: 1. irrigation + high speed 2. no irrigation + low speed. The implants were removed after 4 or 8 weeks. The following were assessed: BIC%, existing bone and soft tissue in contact with the implant.	The amount of new bone after 8 weeks was greater than after 4 weeks, regardless of the bed preparation method. Both methods tested gave similar %BIC parameters after 4 and 8 weeks.	Both methods tested have a similar effect on osseointegration.
Yi [49]	To compare hard tissue changes around titanium implants placed immediately after extraction versus after an 8-week healing period.	In 5 beagle dogs, mandibular premolars were extracted for experimental use. The left side assessed bone healing after extraction or site preparation, while the right side compared immediate and 8-week delayed implant placement. Bone regeneration, implant integration, and bone height were analyzed histologically.	Implant bed preparation resulted in greater bone height loss and lower bone area/total area (BATA) than regular extractions. Immediate implants showed more buccal and lingual bone loss compared to delayed implants. However, immediate placement achieved significantly higher bone-to-implant contact (BIC)	Implant bed preparation and immediate placement increased bone height loss, despite better implant integration.
Tabrizi [38]	To determine whether bone viability is affected by speed and depth of drilling.	100 patients requiring mandibular first molar implants were divided into four equal groups with different drilling parameters: 1000 rpm/10 mm, 1500 rpm/10 mm, 1000 rpm/13 mm, and 1500 rpm/13 mm. Collected bone samples underwent histological processing at 200× magnification.	Bone viability was low across all groups, but significantly the lowest was the group with the following parameters: 1500 rpm/13 mm indicating that the combined increase in both speed and depth substantially reduced bone viability.	Increasing either drilling speed or drilling depth alone does not significantly affect bone viability while increasing both parameters significantly reduces viability of bone cells at the implant site.
Rugova and Abboud [58]	To assess the thermal effects and osseointegration outcomes of different dental implant drilling protocols.	54 dental implants were inserted in 5 male sheep. The groups used different drilling techniques: a standard sequential protocol, a modified protocol with a new-generation drill bit, and a one-drill protocol. Two implant types were used. Histological analysis was conducted 3 weeks post-surgery.	The new-generation one-drill protocol generated significantly less heat, the one-drill protocol remained below 50 °C at all drilling depths, while the sequential drilling protocol consistently exceeded 50 °C, indicating potential thermal damage to bone tissue. Simplified drilling protocols showed slightly higher BIC compared to the traditional approach. The duration of the modified protocol was 2–4 min while the traditional protocol was 12 min.	Simplified drilling protocols using new-generation drill bits can reduce procedure time while maintaining comparable bone-to-implant contact.
Chen [65]	Assessment of implant healing and osteogenesis using a new preparation technique.	New drill design with low speed were used to prepare beds for implants.	This method reduced tissue overheating due to lower speed of preparation,	This method efficiently cuts bone at low rotational speeds, eliminating the need for irrigation while preserving osteocyte viability and accelerating bone reconstruction.
Kosior [44]	Microscopic analysis of the level of bone tissue deformation after drilling under variable conditions in three dental implant system—Straumann, Osstem and S-wide.	27 samples—3 dental implant systems, 3 speeds: 800, 1200, 1500 rpm and 3 types of cooling—without irrigation; with using physiological saline at room temperature; with physiological saline at low temperature were used for this study.	All drilling systems showed satisfactory results when the rotational speed was kept below 800 rpm, regardless of the cooling temperature.	The Straumann system provides the optimal bone surface structure for successful osseointegration.
Pantani [52]	To investigate the influence of lateral pressure exerted on the implant bed as well as osseointegration and changes at the bone level.	In 6 Labradors, bilateral premolars were removed and first molars were hemisectioned. After 3 months, 2 implants of 3.75 mm diameter and 8.5 mm length were implanted on one side using a 3 mm diameter drill as the last. On the other side, the last drill was 2.8 mm in diameter. The dogs were euthanized after 4 months and a histopathological examination was performed.	All implants were integrated with mineralized bone, well embedded in a mature bone plate in the cortical compartment, the apical part of the implant was surrounded by bone marrow. The alveolar process was resorbed in both the premolar and molar regions.	In the case of conventional implant placement, lateral pressure on the walls of the implant bed has no influence on the osseointegration process.
Eom [45]	To investigate the influence of the implant and drill diameter on the implant stability and bone response.	In 10 pigs, the premolars and first molars of the mandible were removed. After 8 months, implants of 3.5 mm in diameter and 8.5 mm in length were implanted using different diameter end drills. Group I: 3 mm, group II: 3.3 mm, group III: 3.5 mm. One animal was killed immediately, the others after 1, 3 or 5 weeks.	In groups I and II, microcracks and collagen deformation were visible in the bone surrounding the implant. In group I, bone resorption reached 1.2 mm, and in group III, up to 0.5 mm.	Bone resorption and fibrous tissue formation occur when an implant is placed in overloaded bone and microcracks develop.
Al-Marshood [53]	To investigate the osseointegration of dental implants implanted in the mandible of dogs using the conventional or undersized technique.	Lower premolars were removed from 12 Beagle dogs. After 3 months, 4.1 mm diameter × 8.5 mm long implants were placed using a 3.8 mm diameter drill in group I and a 3.3 mm diameter drill in group II. The dogs were euthanized 3 months later.	In group II, the implant-to-bone contact occurred at or above the first screw thread of the implant. In half of the implants in group I, the first screw thread was not covered with bone tissue but with fibrous tissue. The bone-to-implant contact percentage (BIC%) was higher in group II.	Preparing the implant bed to be too small may improve the bone response to the implant.

**Table 2:** Detailed characteristics of the studies.

Authors	Animal Model/Tissue Type/Samples Number	Site of Implant Placement/Bed Preparation	Implant Bed Preparation Methods/Surgical Technique	Healing Time	Results of Histological Analysis	Bone-to-Implant Contact (BIC%)
Nkenke [39]	9 minipigs, 6 implant site preparations each	Maxilla	Spiral drill Osteotome	0, 1, 2, 3, 4 or 5 months	No data.	Osteotome: 0 months: buccal-82 ± 7, palatal-79 ± 7 1–3 months: buccal-87 ± 10, palatal-82 ± 24 4–5 months: buccal-72 ± 22, palatal-75 ± 16 Spiral drills: 0 months: buccal 79 ± 6, palatal-59 ± 30 1–3 months: buccal-58 ± 31, palatal-53 ± 26 4–5 months: buccal-77 ± 22, palatal-76 ± 18
Trisi [54]	5 sheep bone, 20 implant site preparations	Mandible	Drill sequence 3.2 to 3.9 mm	2 months	A (no irradiation)—complete bone resorption, no implant integration, necrosis, sequestrum, clearly visible inflammatory infiltrate. B (internal irradiation)—integration of all implants, noticeable bone remodeling, small defects present. C (external irradiation)—noticeable new bone formation and its remodeling, good integration of implants with bone. D (internal and external irradiation)—large areas of bone resorption present, noticeable inflammatory cells, areas integrated with preserved bone present.	Group A 15.068 ± 10.07 Group B 44.02 ± 8.29 Group C 41.198 ± 13.48 Group D 31.024 ± 8.21
Trisi [55]	2 sheep, 20 implant site preparations	Iliac crest	-Drill (1.8 mm) -control group -Drill (3.2 mm) -test group	2 months	Control group: Thread areas not covered by bone are present. Tested group: Newly formed continuous bone layer around the implant is present; osteocorticization phenomenon is noticeable.	Test group: 70.91 ± 7.95 Control group: 49.33 ± 10.73
Kesler [66]	36 rats, 2 implant site preparation each	Tibia	-Er:YAG laser (Opus 20; Lumenis, Nowy Jork, NY) -Drill (1.3 mm)	3 weeks and 3 months	After 3 weeks: The test group (laser) showed more new bone formation in contact with the implant than the control group (drill). After 3 months: Both groups showed the presence of new trabecular bone.	3 weeks: laser: 59.48 control: 12.85 3 months: laser 73.54 control 32.65
Fujiwara [47]	12 dogs, 2 implant site preparation each	Mandible	-Drill (3 mm)	4 and 8 weeks	In both groups, there was noticeable new bone growth after 4 and 8 weeks.	After 4 weeks: Low speed: 46.6 ± 7.3 High speed: 43.1 ± 6.8 After 8 weeks Low speed: 60.0 ± 11.1 High speed: 60.2 ± 6.2
Fujiwara [48]	12 dogs, 2 implant site preparation each	Mandible	-Piezoelectric device -Drill	4 and 8 weeks	In both groups, new bone, osteoid tissue and marrow structures were present after 4 and 8 weeks. After 4 weeks, bone remnants were present in the drill group.	After 4 weeks piezoelectric: 54.9 ± 6.7 drill: 49.5 ± 14.4 After 8 weeks: piezoelectric: 67.4 ± 6.7 drill: 62.9 ± 12.5
Semez [62]	6 sheep (female), 108 implants (18 implants per sheep, 9 implants in each side of the pelvis)	Iliac bones of the pelvis	Er:YAG laser osteotomy Piezoelectric Drill	4, 6 and 8 weeks	No signs of peri-implant hard and soft tissue inflammation or infection were found in any technique. Laser and piezoelectric technique is comparable to drill osteotomy concerning early osseointegration.	After 4 weeks: Laser = 74.51 (4.03) Piezoelectric = 75.14 (13.59) Drill = 72.92 (14.28) After 6 weeks: Laser = 52.85 (13.45) Piezoelectric = 52.92 (19.84) Drill = 75.97 (10.73)
Schierano [42]	15 male Wistar rats, 2 implant sites each	Tibia	Piezoelectric osteotomy Drill	30 days	Newly formed bone had similar characteristics to the original bone in both groups. Some areas of fibrosis were observed around all implants. Some areas exhibited mature Haversian matrix deposition constituting cortical and cancellous bone. Piezosurgery had higher values for bone area with the threads and proportion of mineralized tissue.	Drill = 80.42 ± 10.88 Piezo = 70.25 ± 16.93
Cesaretti [51]	6 Labradors × 4 implants	Mandibule, premolar and molar area	- Drill 3 mm (control) - Drill 2.8 mm Implant size 3.75 × 8.5 mm	4 months		Premolar: 59 (14.7) Premolar control: 48.6 (8.8) Molar: 51.7 (16.2) Molar control: 54.1 (15.1)
Zizzari [59]	10 pigs × 6 implants	Mandible	- Drill 3 mm (G1) - Drill 3.3 mm (G2) - Drill 3.5 mm (G3) Implant size 3.5 × 8.5 mm	0, 1, 3 or 5 weeks	In G1 and G2 there was a collagen deformation and microcracks.	Immediately: G1 = 85.5 ± 2.6 G2 = 56.0 ± 7.4 G3 = 0 ± 0 At week 5: G1 = 80.0 ± 11.6 G2 = 73.2 ± 18.1 G3 = 66.6 ± 14.9
Zeitouni [60]	12 Beagle dogs × 4 implants	Mandible	- Drill 3.8 mm (G1) - Drill 3.3 mm (G2) Implant size 4.1 × 8.5 mm	3 months	In 50% of implants from G1 the first screw-thread was not covered with the bone.	G1 = 47.5 (11.7) G2 = 60.6 (14.2)
Yi [49]	5 male sheep, 12 implant sites per each	Mandible	Control group (sequential protocol) Group A (modified protocol) Group B (one-drill protocol)	3 weeks	Control group—irreversible tissue damage with immediate cell death.	No numeric data
Semez [41]	4 Beagle dogs, 6 implant sites per animal	Mandible	Er:YAG laser osteotomy or drill	2 or 12 weeks	The Er:YAG laser osteotomy resulted in wider peri-implant gaps. No signs of thermal side effects in either group. At 12 weeks: Ongoing undisturbed formation of new bone in both groups, with implants surrounded by mature lamellar bone.	No numeric data
Trisi [56]	100 human	Mandible at the first molar site	Drill system	No data	All groups showed a low level of bone viability. Group: 1500 rpm, 13 mm significantly reduced the percentage of viable cells compared to all other groups.	No data
Stelzle [40]	3 male adult minipigs, 4 implant sites per animal	Tibia	Magneto-dynamic mallet Drill	0 or 14 days	In mallet sites compared to drill sites: Increase in newly formed bone, increase in bone percentage, increase in osteoblast number, smaller amount of fibrous tissue, lateral bone condensation (osseocondensation).	No data
Scarano [61]	10 bovine bone, 100 implant site preparations	Rib	- Piezoelectric system - Drill (2 × 13 mm)	No data	Drill method: Bone chips; irregular bone surface; microcracks, osteocytes with damage Piezoelectric method: No microcracks; bone structure has greater regularity; osteocytes without damage	No data
Stubinger [57]	6 Beagle dogs 12 implant site preparations	Mandible	Dill or piezo	8 weeks	When the original position of the bony crest was taken into account, a higher bone loss and a more apical position of the peri-implant mucosa resulted at the test sites.	No data
Tabrizi [38]	6 sheep 24 implant site preparation	Illac crest	Piezo or drill	15 or 30 days	Piezoelectric—more rapid healing, more organized newly formed bone.	No data
Schierano [43]	Sheep	Femoral heads	Laser Er:YAG Drill 3 mm	0	Laser cutting preserves the trabecular structure at the margin cuts unlike conventional drilling.	No data
Schwarz [50]	6 rabbits 36 implant site preparation	Tibia	Drill: 1.5 mm, 2.0 mm, 2.5 mm, 3.5 mm Low speed 50 rpm without irrigation vs. 800 rpm with irrigation	0	No histological differences between the two surgical drill technique in optical microscope.	No data
Sirolli [64]	18 rats	Maxillary	Drill (1, 1.3 or 1.6 mm) New type of drill (1.0 or 1.6 mm)	0.5/3/7 days	Less heat damage caused by new type of drill. Higher osteoclast marker TRAP after using conventional drill.	No data
Rugova and Abboud [58]	Pigs 27 boreholes	Porcine ribs	Drill (systems: Osstem 3.0 mm, Straumann 3.3 mm, S-wide 5.5 mm)	0	Osstem—smooth surface, no damage to surrounding tissues, bone structure unchanged, small chips, partial compression of the bone, signs of mild carbonization. Straumann—smooth surface, uneven drill holes, no signs of carbonization. Adjacent tissue without damage. S -wide—uneven but smooth surface without damage to adjacent tissues.	No data
Gaspar [63]	4 pigs × 10 implants	Tibia	- Drill 3 mm - Piezoelectric system (Piezosonic Driller^®^) Drills: ESO 18D, ESO 19D, ESO 40A, ESO 40B, oscillating 27–31 Khz, power 50 W	2, 7, 14 or 28 days	At 2 days: In rotary group smooth surface, in piezoelectric group irregular pattern. At 7 days: Greater osteogenesis in the rotary group. At 14 days: In piezoelectric group diffuse new bone formation, in rotary group intense bone formation with greater quantity and better organization. At 28 days: Mature bone in both groups.	No data

**Table 3:** Comparison of the implant bed preparation techniques.

Technique	Osseointegration Rate/Earliest Observed Signs of Healing	Bone Structure After Healing	Technique Sensitivity	Advantages	References
Rotary Drill	Moderate—slower integration/after 3–4 weeks	Moderate new bone formation, but often with more irregularities and delayed organization	Low	Versatile and easy to control	[38,41,44,45,47,48,49,52,53,55,61,63,65,66]
Piezoelectric Surgery	Good, especially in cancellous bone/14–30 days	High osteoblast activity, early bone matrix formation, smooth osteotomy surfaces	High	Minimally invasive, supports osteogenesis	[40,42,46,48,51,57,59,61,64]
Er:YAG Laser	Variable/ 14–21 days	Early woven bone formation with minimal thermal effects and preserved bone structure	High	Minimal necrosis, no thermal damage	[40,41,50,57,60,62,66]
Osteotome	Fast during early healing/within 1 month	Dense and well-adapted Higher BIC in first 1–3 months, denser bone near implant threads	Moderate	Improved stability in soft bone	[39]
Magneto-dynamic Mallet	Very rapid/ 14 days (T14)	High osteoblast activity Significantly more newly formed bone, higher osteoblast count, less fibrous tissue	High	Accelerated healing, less fibrous tissue	[43]
Osseodensification	Moderate/by 60 days	Dense, granular microstructure Granular new bone formation, higher bone density despite similar BIC compared to drilling	Moderate	Increased density and primary stability	[56]

**Table 4:** Quality assessment—JBI checklist for quasi-experimental studies (non-randomized experimental studies).

Authors	1. Is It Clear in the Study What Is the “Cause” and What Is the “Effect”?	2. Were the Participants Included in Any Comparisons Similar?	3. Were the Participants Included in Any Comparisons Receiving Similar Treatment/Care, Other than the Exposure or Intervention of Interest?	4. Was There a Control Group?	5. Were There Multiple Measurements of the Outcome Both Pre and Post the Intervention/Exposure?	6. Was a Follow up Completed, and If Not, Were the Differences Between Groups in Terms of Their Follow up Adequately Described and Analyzed?	7. Were the Outcomes of the Participants Included in Any Comparisons Measured in the Same Way?	8. Were the Outcomes Measured in a Reliable Way?	9. Was Appropriate Statistical Analysis Used?
Nkenke [39]	Yes	Yes	Yes	No	Yes	Yes	Yes	Yes	Yes
Scarano [61]	Yes	Yes	Yes	Yes	No	Yes	Yes	Yes	Yes
Trisi [54]	Yes	Yes	Yes	No	Yes	Yes	Yes	Yes	Yes
Trisi [55]	Yes	Yes	Yes	Yes	Yes	Yes	Yes	Yes	Yes
Kesler [66]	Yes	Yes	Yes	Yes	Yes	Yes	Yes	Yes	Yes
Fujiwara [47]	Yes	Yes	Yes	Yes	Yes	Yes	Yes	Yes	Yes
Fujiwara [48]	Yes	Yes	Yes	Yes	Yes	Yes	Yes	Yes	Yes
Semez [62]	Yes	Yes	Yes	Yes	No	Yes	Yes	Yes	No
Trisi [56]	Yes	Yes	Yes	Yes	Yes	Yes	Yes	Yes	Yes
Stelzle [40]	Yes	Yes	Yes	Yes	No	Yes	Yes	Yes	Yes
Semez [41]	Yes	Yes	Yes	Yes	Yes	Yes	Yes	Yes	No
Schierano [42]	Yes	Yes	Yes	No	Yes	Yes	Yes	Yes	Yes
Yi [49]	Yes	Yes	Yes	Yes	Yes	Yes	Yes	Yes	Yes
Stubinger [57]	Yes	Yes	Yes	Yes	Yes	Yes	Yes	Yes	Yes
Tabrizi [38]	Yes	Yes	Yes	No	No	Yes	Yes	Yes	Yes
Schierano [43]	Yes	Yes	Yes	Yes	Yes	Yes	Yes	Yes	Yes
Schwarz [50]	Yes	Yes	Yes	Yes	Yes	Yes	Yes	Yes	Yes
Sirolli [64]	Yes	Yes	Yes	Yes	Yes	Yes	Yes	Yes	Yes
Rugova and Abboud [58]	Yes	Yes	Yes	Yes	Yes	Yes	Yes	Yes	Yes
Cesaretti [51]	Yes	Yes	Yes	Yes	Yes	Yes	Yes	Yes	Yes
Zizzari [59]	Yes	Yes	Yes	Yes	Yes	Yes	Yes	Yes	Yes
Zeitouni [60]	Yes	Yes	Yes	Yes	No	Yes	Yes	Yes	Yes
Gaspar [63]	Yes	Yes	Yes	Yes	No	Yes	Yes	Yes	Yes
Chen [65]	Yes	Yes	Yes	Yes	Yes	Yes	Yes	Yes	Yes
Kosior [44]	Yes	Yes	Yes	Yes	No	Yes	Yes	Yes	Yes
Pantani [52]	Yes	Yes	Yes	Yes	Yes	Yes	Yes	Yes	Yes
Eom [45]	Yes	Yes	Yes	Yes	Yes	Yes	Yes	Yes	Yes
Al-Marshood [53]	Yes	Yes	Yes	Yes	Yes	Yes	Yes	Yes	Yes
Sakita [46]	Yes	Yes	Yes	Yes	Yes	Yes	Yes	Yes	Yes

## Data Availability

Data supporting the findings of this study are available within the article.
